# Editorial: Impacts of cytoskeletal dysregulation on spinogenesis

**DOI:** 10.3389/fncel.2026.1999766

**Published:** 2026-09-28

**Authors:** Keerthana Surabhi, Erzsebet M. Szatmari, Robert M. Hughes

**Affiliations:** 1 Department of Anatomy and Cell Biology, Brody School of Medicine, East Carolina University, Greenville, NC, United States; 2 Department of Chemistry, East Carolina University, Greenville, NC, United States

**Keywords:** actin, cytoskeleton, dendritic spine, neurodegenerative disease, optogenetics, synaptogenesis

Dendritic spines are highly dynamic morphological features of excitatory neurons in the mammalian brain that form the postsynaptic component of most excitatory synapses and are therefore critical for synaptic plasticity, learning, and memory formation (Hering and Sheng, 2001; Runge et al., 2020). Cytoskeletal remodeling is closely tied to spine dynamics, including new spine formation (spinogenesis), maturation and stabilization of existing spines, and spine loss/elimination (Sala and Segal, 2014). Disrupted cytoskeletal dynamics can lead to abnormal spine morphology and structural plasticity, setting the stage for neurodegeneration (Ammassari-Teule, 2020; Bhembre et al., 2025). The articles in this Research Topic provide evidence for a link between cytoskeletal anomalies and neuronal dysfunction, using a range of approaches, including mass spectrometry-based studies, studies in cultured neurons, and animal models.

Lloyd and Litwa review the interaction between N-methyl-D-aspartate (NMDA) receptor-mediated signaling and remodeling of dendritic spine cytoskeleton during synaptic development. In the mammalian brain, the heterotetrameric NMDA receptors are composed of obligatory GluN1 and regulatory GluN2 subunits. Cytoskeletal-regulating proteins are key regulators of the transition from GluN2B-containing receptors in the developing brain to postnatal GluN2A enrichment. Importantly, the functional difference between these subunits extends beyond their ion-channel properties, as GluN2A and GluN2B exhibit distinct interactions with actin-regulatory proteins involved in spine structural and functional plasticity. The review also discusses abnormal changes in GluN2 isoform expression that disrupt actin-driven spine dynamics, contributing to the pathomechanism of neurological disorders (Myers et al., 2019).

A review by Matsubayashi and Takano describes novel techniques for elucidating the molecular composition of individual synapses with high spatial resolution. Proximity labeling approaches such as BioID and APEX combine localized protein labeling with mass spectrometry to overcome the spatial and cell-type-specific limitations of conventional proteomics methods. Using this technique in the context of spine cytoskeletal dynamics, it has been shown that *C*apping protein, *Ar*p2/3, *M*yosin*IL*inker 3 (CARMIL3), a previously uncharacterized protein, interacts with the synaptic cytoskeletal regulator protein WAVE-associated Rac GTPase-activating protein (WRP) and neuronal actin capping protein during spinogenesis, contributing to spine maturation and synapse unsilencing (Spence et al., 2019). Moreover, these techniques enable the evaluation of the synaptic proteome at the sub-compartmental level. Using synaptopodin-fused BioID2 in the mouse brain, it has been revealed that Pdlim7, an actin-binding protein in the spine apparatus, coassembles with synaptopodin and actin to regulate spine structure and function (Falahati et al., 2022). Proximity labeling is a powerful technique for evaluating changes in the synaptic proteome in healthy and diseased brains.

Mann et al. evaluated how alterations at the actin-ATP interface contribute to cytoskeletal abnormalities associated with neurodegeneration. Under cellular stress with reduced ATP availability, actin bound to ADP has higher affinity for cofilin and can promote cofilin-actin rod formation. The authors examined mutations in the nucleotide-binding residues using the stress- and light-responsive CofActor system. Several of these mutations, such as K18A, D154A, G158L, and K213A, promoted the formation of abnormal actin-rich structures resembling cofilin-actin rods and Hirano bodies, structures associated with neurodegenerative diseases (Bamburg and Bernstein, 2016). The authors show that neurons carrying the K18A and D154A mutations have substantial actin aggregation and reduced spine density compared with neurons expressing wild-type actin. These findings provide a direct link between molecular perturbations in actin and loss of dendritic spines, a critical early event in neurodegeneration.

Neuronal activity promotes cytoskeletal remodeling associated with dendritic spine development (Hotulainen and Hoogenraad, 2010; Hu et al., 2008). López-Ibarra et al. investigated hippocampal neurogenesis in the context of hyperexcitability, including spinogenesis in newly generated neurons and whether these processes alter hippocampus-dependent spatial memory. The authors report that severe hyperexcitability alters neuronal positioning and increases dendritic spine density in adult-born granule cells, whereas milder hyperexcitability enlarges the presynaptic bouton. Despite these neuronal compartment-specific effects, both levels of hyperexcitability impaired spatial location recognition while sparing contextual fear memory.

In the healthy brain, Microtubule-associated protein (MAP) tau is localized mainly to axons, where it stabilizes microtubules and supports axonal transport. Tau hyperphosphorylation reduces its association with microtubules and promotes pathological aggregation, which is a hallmark feature of neurodegeneration, including in Alzheimer's disease (Hernández and Avila, 2007; Weingarten et al., 1975). Hoegy et al. review the involvement of small GTPases in various stages of tau pathology, including phosphorylation, aggregation, propagation, and clearance. Members of the Ras superfamily—including Ras, Rho, Rab, Arf, Ran, and Miro—regulate processes ranging from cytoskeletal dynamics to membrane and vesicular trafficking. Dysregulation of small GTPase signaling, including Rab, Rho, and Ras, following amyloid exposure has been shown to perturb tau homeostasis, leading to tau hyperphosphorylation and aggregation (Kiral et al., 2018). The authors propose that modulating small GTPase signaling may offer therapeutic opportunities for tauopathies.

In summary, this Research Topic is a comprehensive genes-to-behavior overview of the cytoskeletal remodeling in dendritic spine dynamics in health and disease. The articles in this Research Topic include a review of novel techniques to elucidate the composition of the synaptic proteome; two reviews of the signaling mechanisms connecting dendritic spine remodeling with NMDA receptor function and small GTPase signaling; an original article investigating the role of point mutations in the ATP-binding domains within actin in the formation of neurodegenerative cellular inclusions; and finally an article showing differential effects of high and moderate hyperexcitability on the synaptic morphology and integration of newly generated neurons in the hippocampus, both leading to impaired spatial memory. A better understanding of the complex interplay between cytoskeletal remodeling and spine dynamics across the lifespan should help identify therapeutic targets for neurological disorders associated with spine loss and synaptic dysfunction.
